# Prostatic calcifications: Quantifying occurrence, radiodensity, and spatial distribution in prostate cancer patients

**DOI:** 10.1016/j.urolonc.2020.12.028

**Published:** 2021-10

**Authors:** Saurabh Singh, Eleanor Martin, Henry F.J. Tregidgo, Bradley Treeby, Steve Bandula

**Affiliations:** a Centre of Medical Imaging, University College London, London, UK; b Wellcome/EPSRC Centre for Interventional & Surgical Sciences (WEISS), University College London, London, UK; c Department of Medical Physics and Biomedical Engineering, University College London, London, UK

**Keywords:** Prostate MRI, Calcification, HIFU, Radiotherapy, Prostate cancer, PSMA PET

## Abstract

**Background:**

To evaluate the prevalence, density, and distribution of prostate calcification in patients with prostate cancer.

**Methods:**

Patients who underwent both Gallium-68 PSMA PET/CT and MRI of the prostate over the course of a year were selected for analysis. The CT images with visible calcifications within the prostate were included and calcifications automatically isolated using a threshold of 130 HU. The corresponding multiparametric MRI was assessed and the peripheral zone, transition zone, MRI-visible tumor, and urethra manually contoured. The contoured MRI and CT images were registered using rigid registration, and calcifications mapped automatically to the MRI contours.

**Results:**

A total of 85 men (age range 50–88, mean 69 years, standard deviation 7.2 years) were assessed. The mean serum Prostate Specific Antigen PSA was 16.7, range 0.12 to 94.4. Most patients had intermediate-risk disease (68%; Gleason grade group 2 and 3), 26% had high-risk disease (Gleason grade group 4 and 5), and 6% had low-risk disease (Gleason grade group 1). Forty-six patients out of 85 (54%) had intraprostatic calcification. Calcification occurred more in transition zone than the peripheral zone (65% vs. 35%). The mean density of the calcification was 227 HU (min 133, max 1,966 HU). In 12 patients, the calcification was within an MRI-visible tumor, in 24 patients, there were calcifications within a 9 mm distance of the tumor border, and in 9 patients, there were calcifications located between the urethra and tumor.

**Conclusions:**

Calcifications are common in patients with prostate cancer. Their density and location may make them a significant consideration when planning treatment or retreatment with some types of minimally invasive therapy.

## Highlights

• Intraprostatic calcifications are under-recognized and under-reported in imaging.
• Intraprostatic calcifications are common in patients with prostate cancer.
• They commonly occur within tumors or in the vicinity of tumors.

## Graphical abstract

**Figure:**
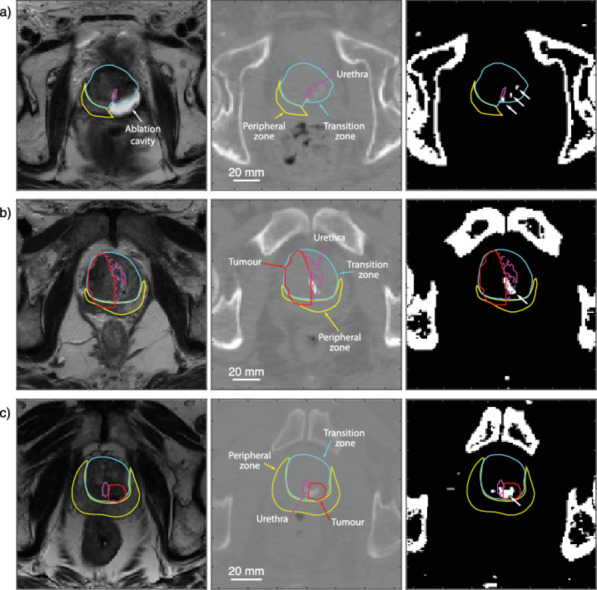

## 1 Introduction

Prostatic calcifications are commonly found in men and are thought to be associated with prostatitis, chronic pelvic pain syndrome, and prostate cancer [1,2]. Previously these calcifications were not considered clinically significant and their presence usually not mentioned in diagnostic imaging reports. Recent studies have however shown that high-density material such as calcification can have a significant impact on treatment delivery in high intensity focused ultrasound (HIFU), transurethral ultrasound ablation, and brachytherapy [3], [4], [5], [6]. In ultrasound therapy, the high-density inclusions can cause reflections of the ultrasound beam, causing changes in the treated volume, which can result in over or under treatment [4,5]. In brachytherapy, the presence of prostatic calcification changes the tissue effective atomic number, leading to altered dose distribution and potential underdosing [3]. Improved understanding of the formation, composition, and distribution of these calcifications could allow development of treatment strategies which mitigate these effects.

The pathogenesis of prostate calcification is thought to be related to prostatic inflammation, urinary retention, or prostatic reflux [7], [8], [9]. These factors are also thought to have a role in prostate cancer and therefore it is not surprising that calcifications coexist in prostates with cancer. The source of calcification is thought to be desquamated acinar cells which form a substance called corpora amylacea. Hydroxyapatite (HA) is then deposited on corpora amylacea forming corporal calculi [7]. An alternative mechanism of HA deposition has been proposed by a group who suggest that HA is deposited by osteoblast-like epithelial cells. The authors suggest that osteoblast-like epithelial cells may be associated with prostate cancer cells and prostate calcification may be a prognostic marker [10].

A few studies have investigated the prevalence of prostate calcification using either imaging or histopathological analysis. A histological study analyzed 298 consecutive whole mount prostate for patients with prostate cancer and found 88.6% contained calcifications [11]. A lower incidence of 58.8% was reported in a study of patients undergoing transrectal sonography who had prostate cancer on biopsy [12]. However, no studies have accurately mapped the location and distribution of calcifications or analyzed their radiodensity using modern CT and MRI. Furthermore, there has been no investigation of calcifications in patients who have undergone treatments such as brachytherapy, HIFU, transurethral ultrasound ablation, or cryotherapy.

The aim of this study was to accurately map and quantify prostatic calcifications using multimodal imaging and computational tools, in a cohort of patients undergoing or having previously undergone treatment for prostate cancer.

## 2 Materials and methods

Ethical approval for this study was granted by the Yorkshire and the Humber Research Ethics Committee (18/YH/0411).

### 2.1 Study cohort

In order to select patients who had contemporaneous CT and MRI imaging data, a consecutive cohort of patients who underwent Gallium-68 PSMA PET/CT and multiparametric MRI (mpMRI; within 6 months of each other) between August 2017 and August 2018 were retrospectively selected. The clinical indications for PSMA PET at our institution are mainly staging of high-risk prostate cancer and assessment of cancer recurrence after treatment. Patients who had a diagnosis of prostate cancer mentioned in the clinical indications were included. Patients who had undergone radical prostatectomy were excluded. Clinical records of selected patients were reviewed for clinical data including PSA and histopathological reports.

### 2.2 Imaging

A total of 85 datasets were obtained from patients who had undergone both Gallium-68 PSMA PET/CT and MR scans. The whole body CT was acquired with 2.5 mm slice thickness and the modal in-plane resolution was 0.98 mm (min 0.98 mm, max 1.37 mm). The mpMRI included T2W small field of view images, high *b*-value diffusion weighted imaging (DWI) image (b1400 or b2000), apparent diffusion coefficient (ADC) map, and dynamic contrast-enhanced images. The modal in-plane resolution for the MR images was 0.39 mm (min 0.35 mm, max 0.78 mm) and the modal slice separation was 3.3 mm (min 3 mm, max 3.85 mm). The CT images for all patients were assessed by a Board-certified Radiologist (S.S.).

### 2.3 Computational analysis

Of these datasets, the prostate and urethra were contoured on the MR images of 45 datasets, chosen as calcifications were evident on visual inspection of the CT scans. The peripheral and transition zone base, midgland, and apex regions of the prostate, as well as the urethra were contoured by a radiologist, slice by slice using Horos (horosproject.org), then exported as .xml files for further use. Signal abnormality considered as suspicious or highly suspicious for tumor (Likert score 4 or 5) was contoured if it corresponded to cancerous regions on biopsy.

Identification of calcifications was automated in order to reduce variability and increase accuracy of the output statistics. Registration of the CT and MR datasets was performed to allow translation of the contours from the MR images to the CT image space [13], where calcifications can be identified by their radiodensity. For identification of the calculi, the CT images were thresholded at 130 HU. This threshold was selected based on published literature and visual inspection to minimize the detection of noise and beam hardening artifact [14,15]. Each of the contours was defined as a search region, in addition to 3 further search regions. The first of these was derived from the tumor region, grown radially by 9 mm, which is the recommended treatment margin for focal therapy in the prostate [16]. Another region was defined as the volume located between the urethra and tumor search regions. The final region was formed from the sum of all search regions. For each of these regions, clusters of voxels with intensities above the threshold were identified computationally.

For each cluster, the coordinates of the centroid, cluster volume, principal axes lengths of an ellipsoid fitted to the cluster, and mean voxel intensities were computed. Any clusters containing a single voxel were rejected as they were usually found to have voxel intensities close to the threshold, and were indistinguishable from noise on visual inspection. The lateral (in-plane) distance of the centroid of each cluster from the centroid of the urethra search region was also calculated, where the centroid of the urethra was calculated from the mean of the in-plane centroid coordinates across all image slices containing the urethra contour. For each patient, mean, minimum, and maximum values of these quantities were calculated for each contoured region of interest. Calcifications spanning multiple regions of interest were counted in each region. Their statistics were computed only for the part of the calcification located inside each of the zones; the statistics of the entire calcification were computed under the total prostate region, provided it lay fully within that region.

## 3 Results

### 3.1 Patient cohort

A total of 85 men (age range 50–88, mean 69 years, standard deviation 7.2 years) were assessed. The mean PSA was 16.7 ng/ml, range 0.12 to 94.4, standard deviation 19.8. All patients had a diagnosis of prostate cancer and for 78 patients biopsy information was available in their electronic health records. In terms of Gleason grade group; 68% had intermediate-risk disease (Gleason grade group 2 and 3), 26% had high-risk disease (Gleason grade group 4 and 5), and 6% had low-risk disease (Gleason grade group 1). Overall Gleason grade is given in Table 1. Forty-eight patients in this cohort had a history of a previous treatment. Sixteen patients had previous HIFU, 16 had previous external beam radiotherapy, 7 had previous brachytherapy, 3 had previous cryotherapy, 1 had previous chemotherapy, and 1 had reversible electroporation. Some patients had more than one treatment type; 2 had external beam radiotherapy and salvage HIFU, 1 had brachytherapy and salvage HIFU, and 1 had HIFU and cryotherapy.

**Table 1:** Summary of patient demographics

Number of patients	85
Median age (y)	70 (50–88)
Mean PSA level (ng/ml)	16.7 (0.12–94.5)
Overall Gleason grade	
3 + 3	5
3 + 4	28
4 + 3	25
3 + 5	1
4 + 4	5
4 + 5	13
5 + 4	1
Previous treatment	48/85

### 3.2 Prostate calcification

**Fig. 1:**
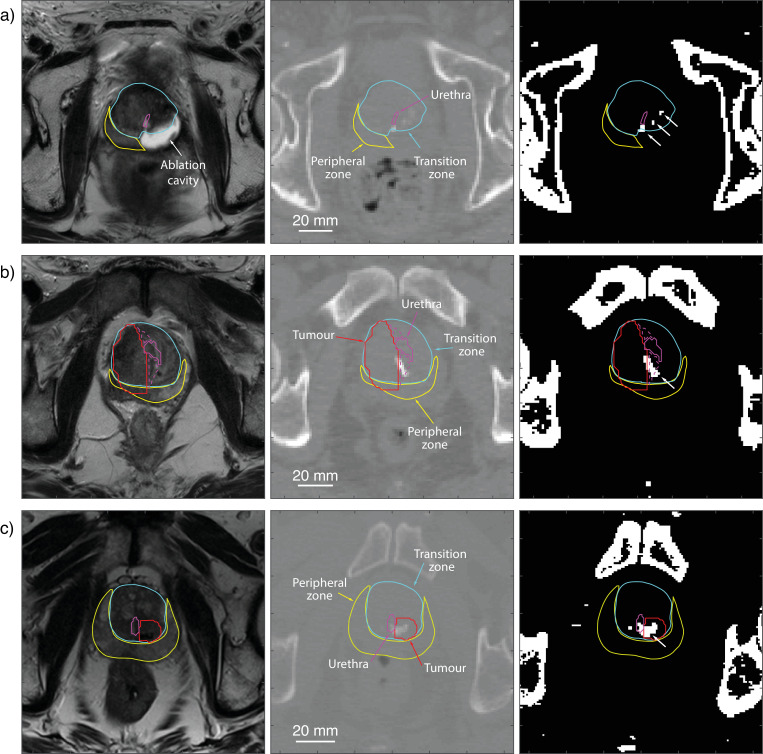
T2-weighted MR image slice (left), the corresponding CT image slice (middle), and thresholded image (right) for 3 example cases: (A) previous left-sided HIFU on which calcifications (highlighted by white arrows) are visible within the transition zone midgland contour (blue). (B) Calcification is visible between the urethra and tumor. (C) Calcification is present within the tumor. The peripheral zone midgland (blue), urethra (purple), tumor (red), and area between urethra and tumor (purple dotted) are also shown. (Color version of figure is available online.)

Intraprostatic calcifications were found in 46 out of 85 patients (Table 2). Examples of corresponding MR and CT images, with visible calculi within transformed region contours, are shown in Fig. 1. An average of 5 foci of calcifications was identified in each patient, with 24 being the highest number of calcifications identified in a single patient. All calcifications were located within 36.9 mm of the centroid of the urethra contour/region of interest (ROI), at a mean distance of 12.1 mm. Calcifications were distributed throughout the regions of the prostate, on average across 4 different regions. The mean volume of calcifications was 55.3 mm^3^. The largest calcification, with a volume of 1,263.6 mm^3^ spanned several ROIs; it is therefore recorded under the “all regions” search volume, whereas for that patient, the largest calcification found in any region individually had a volume of 689.0 mm^3^. This represents only part of the calcification which extends beyond the boundary of this region of interest; a similar occurrence can be observed in Fig. 1A. Most calcifications tended more toward ellipsoid rather than spherical in shape, with a mean aspect ratio of 1:0.74:0.49. Results are shown in for calcifications found in the total region mask and for each ROI individually (Table 2).

**Table 2:** Calcification statistics for all regions together and individually for each contoured region

	Number of patients	Number of calcifications	Urethra distance	Volume	Mean pixel intensity
			mm	mm^3^	HU
All regions	46	5 (1, 24)	12.1 (0.3, 36.9)	55.3 (4.8, 1263.6)	227 (133, 1966)
TZ base	22	2 (1, 12)	10.8 (1.4, 27.4)	40.4 (4.8, 522.1)	251 (133, 1105)
TZ midgland	26	2 (1, 6)	8.6 (1.1, 28.1)	30.4 (4.8, 689.0)	227 (134, 1405)
TZ apex	21	2 (1, 8)	9.1 (2.5, 25.9)	43.1 (4.8, 703.3)	230 (133, 982)
PZ base	9	1 (1, 2)	13.5 (7.3, 21.6)	12.4 (4.8, 40.5)	248 (133, 755)
PZ midgland	18	2 (1, 7)	14.4 (7.0, 22.7)	33.7 (4.8, 376.7)	259 (134, 1966)
PZ apex	20	2 (1, 6)	11.2 (2.6, 22.9)	30.7 (4.8, 191.6)	267 (136, 1292)
Urethra	13	2 (1, 5)	3.6 (0.9, 5.9)	28.1 (4.8, 128.7)	212 (144, 446)
Tumor	12	1 (1, 2)	12.6 (4.7, 31.0)	62.4 (4.8, 326.6)	241 (138, 502)
Tumor margin	24	3 (1, 20)	13.9 (0.3, 36.9)	46.0 (4.8, 439.3)	245 (133, 690)
Between tumor and urethra	9	1 (1, 3)	7.6 (4.3, 15.5)	54.0 (4.8, 327.1)	263 (153, 449)

Number of calcifications, distance from the urethra, volume, and mean pixel intensity are given as mean (minimum, maximum) of the values for each patient. The distance from the urethra is defined as the in-plane straight line distance between the centroid of the calcification and centroid of the urethra region, where the centroid of the urethra is calculated from the mean of the in-plane centroid coordinates across all image slices containing the urethra contour. PZ = peripheral zone; TZ = transition zone.

In 33 out of these 46 patients, a MRI-visible tumor was identified. In 12 patients, there were calcifications within the tumor (see Fig. 1C), and 24 patients had calcifications within a 9 mm border of the tumor (see Fig. 1B). In patients with a previous history of treatment, 24 out of 48 (50%) had prostate calcification. In treatment-naive patients, 22 out of 37 (59%) had prostate calcification. There was no statistically significant difference the 2 groups (*P*= 0.51, Fisher's exact test 2-tailed).

## 4 Discussion

This is the first study to accurately map prostate calcification in patients with prostate cancer using computational methods to analyze rich imaging data available from contemporaneous mpMRI and CT. Our study has shown a few important differences in the distribution of calcification compared to previous studies. Although calcification occurred more in the transition zone, there were a significant number in the peripheral zone (35%), a greater proportion than previously reported, for instance 17% [11] and 6.8% [12].

A higher incidence in the peripheral zone could be explained by differences in the patient cohort studied compared to other studies. This cohort had a high percentage of high-risk disease and history of previous treatment. As the majority of prostate cancers occur in the peripheral zone and calcification can be associated with cancer, this may explain the higher incidence in our cohort. The densities of the calcifications ranged from 133 to 1,966 HU, with a mean of 227 HU. This is comparable to the densities reported in previous studies conducted on smaller patient cohorts [3,17]. The volume of calcifications ranged from 4.8 to 1,263.6 mm^3^. The larger foci were found more in the transition zone, consistent with previous studies.

An important finding of this study is that prostatic calcification can commonly occur within and in the locale of prostate cancer. In 12 patients, foci of calcification were within MRI-visible tumors. The presence of tumor calcification in this cohort is higher than previously observed [11]. This higher incidence may be explained again by many patients in this cohort having previous treatments and then having a local recurrence. We hypothesize that previous treatments such as radiotherapy or thermal ablation cause inflammation resulting in a healing response which leads to deposition of calcification. This process is seen in other organs such as the liver and kidneys [18,19] and has been reported after radiotherapy and thermal ablation in the prostate [20,21]. Furthermore, we found that in 24 patients, there were calcifications within 9 mm of the tumor, which is the ablation margin recommended for focal therapy [16].

The prostatic urethra is an important structure to protect in prostate therapies. Our analysis showed that in 13 patients calcification was in a periurethral distribution, and in 9 patients, calcifications were located between the urethra and tumor (see Fig. 1B). The presence of high-density material such as calcification in the prostate has been shown to cause aberration of ultrasound waves and photon attenuation in radiotherapy. These effects can cause significant differences in treatment dose for both ultrasound ablation and radiotherapy. For transurethral ultrasound ablation, for example, calcifications located around the urethra, between the urethra and tumor, or in the tumor itself, may lie in the path of the beam. If the presence of these calcifications is not accounted for in treatment planning, then there could be a risk of undertreatment and subsequent recurrence. Further studies are needed to model the effects of prostate calcification in treatment delivery in order to determine strategies and robust thresholds for treatment.

MRI has become a key imaging modality in the assessment of prostate cancer but calcifications are difficult to visualize in standard multiparametric protocols [22]. It is widely used to plan thermal ablation treatments such as HIFU, cryotherapy, and transurethral ultrasound ablation. However calcifications are often not visible on MRI and not routinely commented on in radiological reports. Therefore, the impact of calcification in these therapies is likely under-recognized and under-reported. In contrast, for patients undergoing radiotherapy, CT imaging is usually available and calcifications can be used as pseudofiducial markers to plan treatment [23]. However, most CT reports do not mention prostatic calcification, even though studies have shown an impact in dose delivery [3].

## 5 Conclusions

In this study, the location and density of prostatic calcifications were accurately mapped. The study has shown that prostatic calcifications are common in patients with prostate cancer. A large proportion of calcifications occur in and around tumors which could have an impact on their subsequent treatment.

## Conflict of interest

The authors declare that they have no conflicts of interest.

## Appendix Supplementary materials

Image, application 1

## References

[1] Geramoutsos I, Gyftopoulos K, Perimenis P, Thanou V, Liagka D, Siamblis D (2004). Clinical correlation of prostatic lithiasis with chronic pelvic pain syndromes in young adults. Eur Urol.

[2] Shoskes DA, Lee CT, Murphy D, Kefer J, Wood HM. (2007). Incidence and significance of prostatic stones in men with chronic prostatitis/chronic pelvic pain syndrome. Urology.

[3] Fekete CAC, Plamondon M, Martin AG, Vigneault E, Verhaegen F, Beaulieu L. (2015). Calcifications in low-dose rate prostate seed brachytherapy treatment: post-planning dosimetry and predictive factors. Radiother Oncol.

[4] Bakaric M, Martin E, Georgiou PS, Cox BT, Payne H, Treeby BE. (2018). Experimental study of beam distortion due to fiducial markers during salvage HIFU in the prostate. J Therap Ultrasound..

[5] Georgiou P, Jaros J, Payne H, Allen C, Shah T, Ahmed H (2017). Beam distortion due to gold fiducial markers during salvage high-intensity focused ultrasound in the prostate. Med Phys.

[6] Suomi V, Treeby B, Jaros J, Makela P, Anttinen M, Saunavaara J (2018). Transurethral ultrasound therapy of the prostate in the presence of calcifications: a simulation study. Med Phys.

[7] Sfanos KS, Wilson BA, De Marzo AM, Isaacs WB. (2009). Acute inflammatory proteins constitute the organic matrix of prostatic corpora amylacea and calculi in men with prostate cancer. Proc Natl Acad Sci.

[8] Cross P, Bartley C, McClure J. (1992). Amyloid in prostatic corpora amylacea. J Clin Pathol.

[9] Cai T, Tessarolo F, Caola I, Piccoli F, Nollo G, Caciagli P (2018). Prostate calcifications: a case series supporting the microbial biofilm theory. Invest Clin Urol.

[10] Scimeca M, Bonfiglio R, Varone F, Ciuffa S, Mauriello A, Bonanno E. (2018). Calcifications in prostate cancer: an active phenomenon mediated by epithelial cells with osteoblast phenotype. Microsc Res Tech.

[11] Suh JH, Gardner JM, Kee KH, Shen S, Ayala AG, Ro JY. (2008). Calcifications in prostate and ejaculatory system: a study on 298 consecutive whole mount sections of prostate from radical prostatectomy or cystoprostatectomy specimens. Ann Diagn Pathol.

[12] Smolski M, Turo R, Whiteside S, Bromage S, Collins GN. (2015). Prevalence of prostatic calcification subtypes and association with prostate cancer. Urology.

[13] Modat M, Cash DM, Daga P, Winston GP, Duncan JS, Ourselin S. (2014). Global image registration using a symmetric block-matching approach. J Med Imaging.

[14] Bai Y, Wang MY, Han YH, Dou SW, Lin Q, Guo Y (2013). Susceptibility WeightedImaging: a new tool in the diagnosis of prostate cancer and detection of prostatic calcification. PLoS One.

[15] Kucharczyk W, Henkelman RM. (1994). Visibility of calcium on MR and CT: can MR show calcium that CT cannot?. Am J Neuroradiol.

[16] Le Nobin J, Rosenkrantz AB, Villers A, Orczyk C, Deng FM, Melamed J (2015). Image guided focal therapy for magnetic resonance imaging visible prostate cancer: defining a 3-dimensional treatment margin based on magnetic resonance imaging histology coregistration analysis. J Urol.

[17] Hama Y. (2014). Detection of prostate calcification with megavoltage helical CT. Acad Radiol.

[18] Kim YS, Rhim H, Lim HK, Choi D, Lee MW, Park MJ. (2011). Coagulation necrosis induced by radiofrequency ablation in the liver: histopathologic and radiologic review of usual to extremely rare changes. Radiographics.

[19] Wendler J, Porsch M, Hu¨hne S, Baumunk D, Buhtz P, Fischbach F (2013). Short-and mid-term effects of irreversible electroporation on normal renal tissue: an animal model. Cardiovasc Intervent Radiol.

[20] Jones WA, Miller EV, Sullivan LD, Chapman WH. (1979). Severe prostatic calcification after radiation therapy for cancer. J Urol.

[21] Chin JL, Touma N, Pautler SE, Guram KS, Bella AJ, Downey DB (2003). Serial histopathology results of salvage cryoablation for prostate cancer after radiation failure. J Urol.

[22] Bai Y, Wang MY, Han YH, Dou SW, Lin Q, Guo Y (2013). Susceptibility weighted imaging: a new tool in the diagnosis of prostate cancer and detection of prostatic calcification. PLoS One.

[23] Zeng GG, McGowan TS, Larsen TM, Bruce LM, Moran NK, Tsao JR (2008). Calcifications are potential surrogates for prostate localization in image-guided radiotherapy. Int J Radiat Oncol Biol Phys.

