# Supplementary material for: Prostatic calcifications: Quantifying occurrence, radiodensity, and spatial distribution in prostate cancer patients
Source: Urol Oncol. 2021 Oct;39(10):728.e1–6. doi: 10.1016/j.urolonc.2020.12.028 (PMC8492071; doi:10.1016/j.urolonc.2020.12.028)
Supplement: Supplementary file 1 [file mmc1.pdf]

Prostatic calcifications: Quantifying occurrence, radiodensity, and spatial distribution in prostate cancer patients
Supplementary material

**Saurabh Singh · Elly Martin · Henry Tregidgo ·
Bradley Treeby · Steve Bandula**

June 22, 2020

1 Introduction to supplementary material

This supplementary material contains a detailed description of the image registration and image processing methods used to quantify prostatic calcifications in this study. Also included are a set of histograms showing results for all patients: number of calcifications, mean volume of calcifications in each patient, the mean distance of calcifications from the urethra in each patient, the mean aspect ratio of calcifications in each patient, and the mean voxel intensities in each patient.

2 Extended methods

CT and MR image volumes were first converted from DICOM to Nifti format using the dicm2nii Matlab toolbox (1). To account for differing scan coordinates and patient positioning, each registration was first initialised with a manual alignment, performed by visually aligning the hip joints within 2.5 cm in the CT and MR volumes using the NifTK affine transformation module (2). Each MR volume was then rigidly registered to the corresponding CT volume using the manual alignment matrix and the NiftyReg `reg_aladin` function, which uses an intensity-based block-matching algorithm (3). The resulting rigid transformation matrix was then used to resample the MR volumes to match the CT image space.

The remaining image processing was performed in MATLAB (MathWorks, Cambridge, UK). The exported xml contours were first extracted (based on `xml2struct` (4)) and each was fitted with a 100 point spline to obtain a smooth contour. Contours for each region from each image slice were concatenated and converted to a three dimensional binary mask using `blendedPolymask` (5). The cartesian coordinates of the masks in MR image space were obtained and transformed to CT image space by applying the rigid transformation matrix from the image registration. The transformed coordinates were then converted back to binary masks and a slice by slice filling operation was performed to remove any holes in the masks caused by the transformation. The MR images were

Saurabh Singh
Centre of Medical Imaging, 43-45 Foley Street, London, W1W 7TS

acquired with higher resolution than the CT images, leading to discretisation of the contour masks and small deviations in shape from the original contours.

Three further search regions were then formed from the masks. The first of these was derived from the tumour region, grown radially by 9 mm, which is the recommended treatment margin for focal therapy in the prostate (6). The region growing was performed using the `imdilate` function in Matlab, with an arbitrary structure element formed from a sphere of 9 mm radius, which on transformation to the image volume grid coordinates resulted in an ellipsoidal element due to the anisotropic voxel size. The original tumour volume was then subtracted from the mask to enable separate counting of calcifications located within the tumour and within the margin volume. Another region was defined as the volume located between the urethra and tumour search regions. This region was formed by taking the convex hull of the urethra and tumour masks in each image slice. The tumour and urethra masks were then subtracted, and any small groups of pixels were removed to leave a mask which covered only the regions between the urethra and tumour. The final region was formed from the convex hull of the sum of all other search regions.

For identification of calcifications, the CT image volumes were first cropped to include only the slices containing region masks, then thresholded at 130 HU. Above this threshold, only calcified and bony tissue should be visible (7; 8) with minimal image noise on visual inspection. For each ROI in turn, all pixels in the thresholded CT data located outside the ROI mask were set to 0 and the `regionprops3` function was used to obtain details of 3D volumes consisting of groups of voxels with values above the threshold.

3 Results

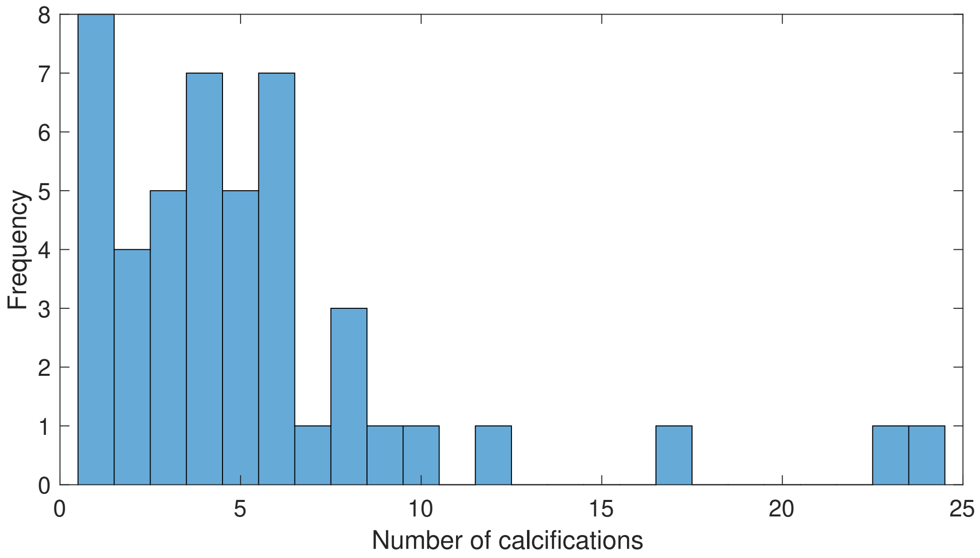

Fig. S1 Distribution of number of calcifications across the patient cohort.

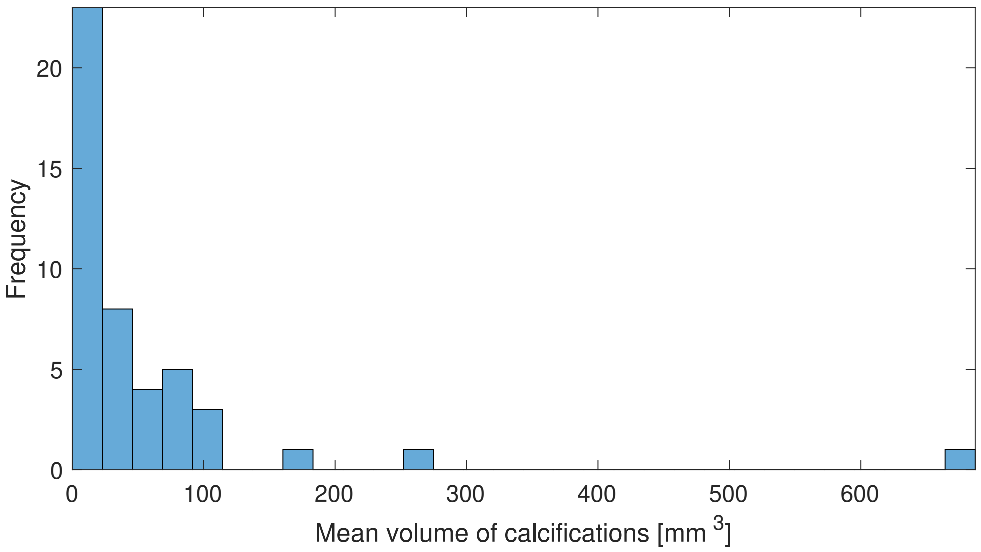

Fig. S2 Distribution of mean volume of calcifications across the patient cohort.

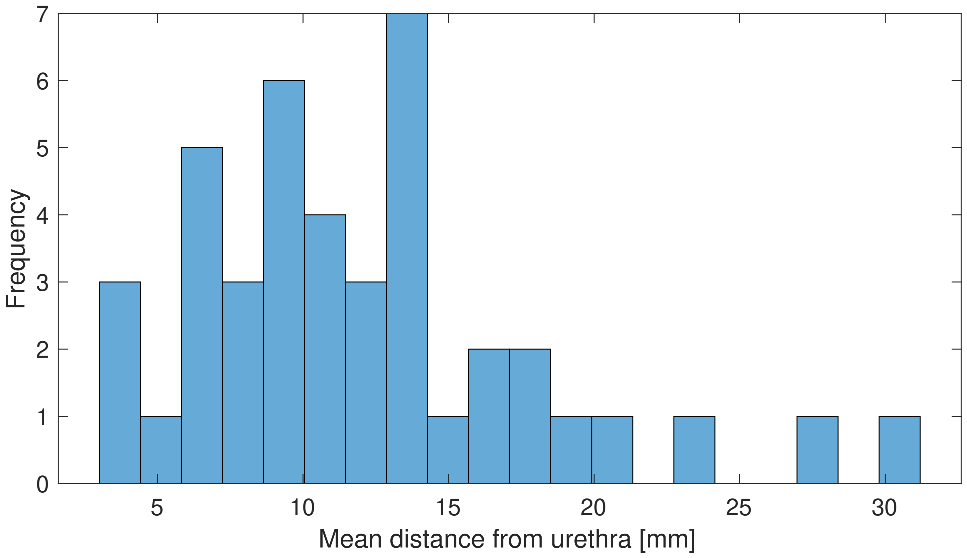

Fig. S3 Distribution of mean distance of the centroid of each calcification from the centroid of the urethra contour.

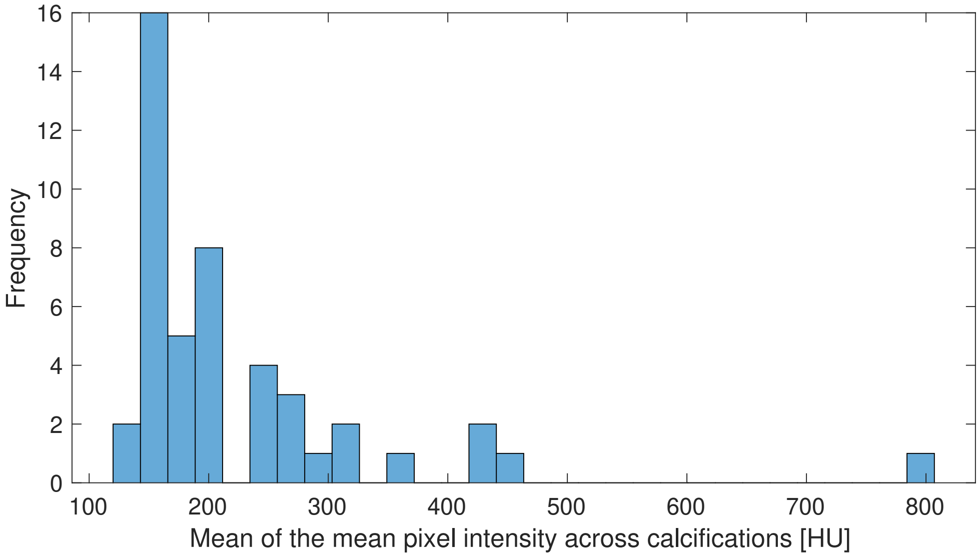

Fig. S4 Distribution of the mean voxel intensity of calcifications across the patient cohort.

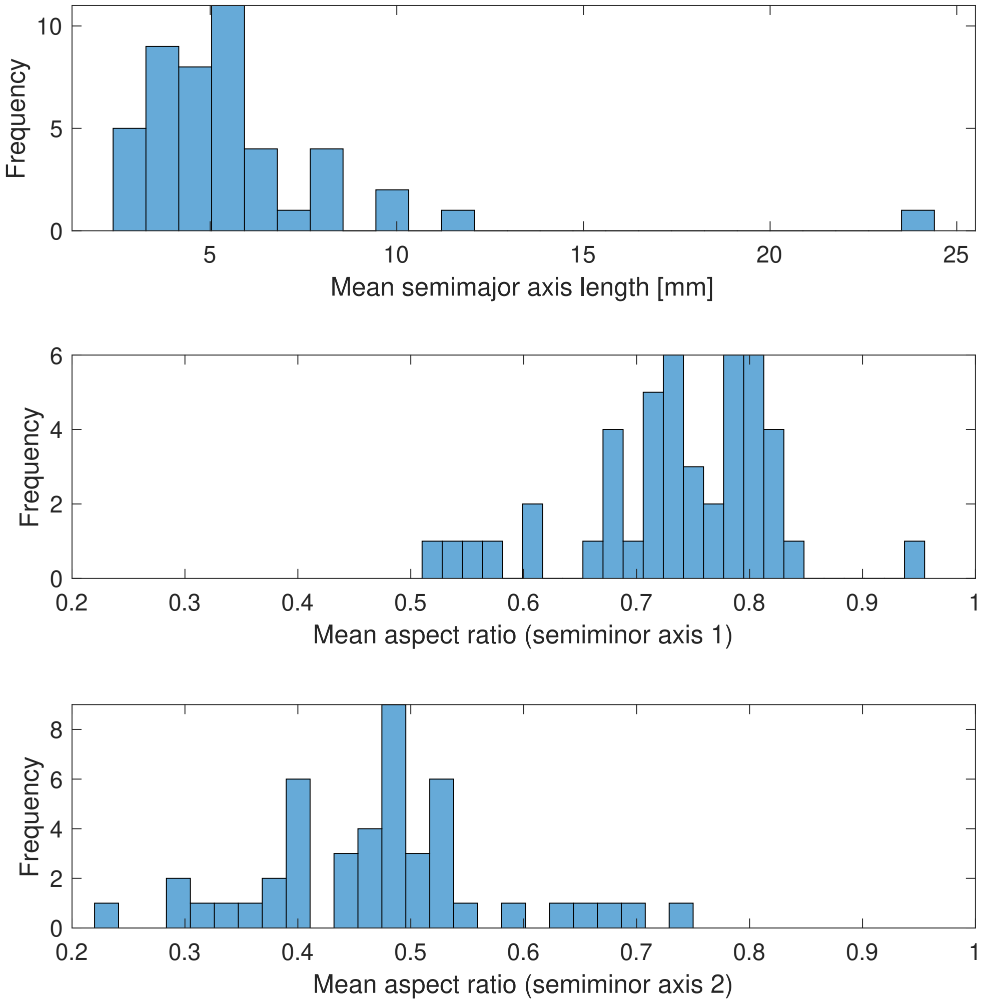

Fig. S5 Distribution of mean principal axis length and aspect ratio of calcifications across the patient cohort.

References

1. Li X, Morgan PS, Ashburner J, Smith J, Rorden C. The first step for neuroimaging data analysis: DICOM to NIfTI conversion. Journal of Neuroscience Methods. 2016;264:47–56.
2. Clarkson MJ, Zombori G, Thompson S, Totz J, Song Y, Espak M, et al. The NifTK software platform for image-guided interventions: platform overview and NiftyLink messaging. International Journal of Computer Assisted Radiology and Surgery. 2015;10(3):301–316.
3. Modat M, Cash DM, Daga P, Winston GP, Duncan JS, Ourselin S. Global image registration using a symmetric block-matching approach. Journal of Medical Imaging. 2014;1(2):1–6.
4. Yeh CY, Mo X, Smirnov I, Wanner A, Falkena W. xml2struct, with bug fix and added features; 2019. MATLAB Central File Exchange. Available from: https://uk.mathworks.com/matlabcentral/fileexchange/58700-xml2struct-with-bug-fix-and-added-features.
5. Holcombe S. Blended 3D poly2mask; 2019. MATLAB Central File Exchange. Available from: https://www.mathworks.com/matlabcentral/fileexchange/37863-blended-3d-poly2mask.
6. Le Nobin J, Rosenkrantz AB, Villers A, Orczyk C, Deng FM, Melamed J, et al. Image guided focal therapy for magnetic resonance imaging visible prostate cancer: defining a 3-dimensional treatment margin based on magnetic resonance imaging histology co-registration analysis. The Journal of Urology. 2015;194(2):364–370.
7. Bai Y, Wang MY, Han YH, Dou SW, Lin Q, Guo Y, et al. Susceptibility Weighted Imaging: A New Tool in the Diagnosis of Prostate Cancer and Detection of Prostatic Calcification. PLOS ONE. 2013 01;8(1):1–7.
8. Kucharczyk W, Henkelman RM. Visibility of calcium on MR and CT: can MR show calcium that CT cannot? American Journal of Neuroradiology. 1994;15(6):1145–1148.
